# Comprehensive theoretical study of the effects of facet, oxygen vacancies, and surface strain on iron and cobalt impurities in different surfaces of anatase TiO_2_

**DOI:** 10.1038/s41598-024-74423-3

**Published:** 2024-10-30

**Authors:** Danil W. Boukhvalov, Vladimir Yu. Osipov, Anna Baldycheva, Benjamin T. Hogan

**Affiliations:** 1 College of Science, Institute of Materials Physics and Chemistry, Nanjing Forestry University, Nanjing, 210037 P. R. China; 2 Institute of Physics and Technology, Ural Federal University, Yekaterinburg, 620002 Russia; 3 Ioffe Institute, St. Petersburg, 194021 Russia; 4 STEMM Laboratory, University of Exeter, Exeter, EX4 4QF UK; 5 Department of Chemical Engineering, Queen’s University, Kingston, K7L 3N6 Canada

**Keywords:** Titanium dioxide, Hydrogen evolution reaction, Transition metal surface impurities, Oxygen vacancies, Catalysis and photocatalysis, Materials for energy and catalysis, Theory and computation

## Abstract

We report the results of systematic ab initio modelling of various configurations of iron and cobalt impurities embedded in the (110), (101), and (100) surfaces of anatase TiO_2_, with and without oxygen vacancies. The simulation results demonstrate that incorporation into interstitial voids at the surface level is significantly more favourable than other configurations for both iron and cobalt. The calculations also demonstrate the crucial effect of the facet as well as the lesser effects of other factors, such as vacancies and strain on the energetics of defect incorporation, magnetic moment, bandgap, and catalytic performance. It is further shown that there is no tendency towards the segregation or clustering of impurities on the surface. The calculated free energies of the hydrogen evolution reaction in acidic media predict that iron impurities embedded in the (101) surface of anatase TiO_2_ can be a competitive catalyst for this reaction.

## Introduction

Transition metal (TM) doped titanium dioxide nanoparticles are gaining attention as prospective materials for photonics and catalysis^1–9^. In particular, photocatalytic applications can be boosted by the decreased bandgap resulting from the inclusion of the metal impurities which can lead to increased light harvesting efficiency^8,10–17^. However, improvements in the catalytic/photocatalytic performance are also reported without any decrease in the bandgap^18,19^. The results of extended X-ray adsorption fine structure measurements demonstrate under-coordination of TM-centres in TiO_2 _nanoparticles^20–22^. The catalytic/photocatalytic properties have also been associated with metal centres located in the surface layer or directly on the surface^23–27^. The metal dopant content in TiO_2 _nanoparticles can be varied smoothly from around 25 wt%^28^down to a few or even half of a weight%^10–12,15,17,18,25–27,29,30^. The photocatalytic activity of TiO_2 _is also associated with electronic processes occurring on the surface in a layer up to 5 nm thick^31^. The catalytic (we will not further use the prefix “photo-” every time when talking about the photocatalytic activity of undoped and metal-doped titanium dioxide) efficiency of TiO_2 _nanoparticles with less than 1 wt% of TM dopants, as reported in multiple works^32–39^, can be associated with the catalytic efficiency of metal impurities in the surface layer as the uniform distribution of such small amounts of impurities within the whole body of nanoparticles should have an insignificant effect on the overall properties of the system. The influence of the surface morphology on the catalytic properties of TiO_2 _nanoparticles is also indirect evidence for surface-located metal impurities as the chemically active part of the system^39,40^. Another piece of indirect evidence for the location of metal impurities on the surface is the 2+ oxidation states observable for the TM centres^20,37^. The incorporation of substitutional metal impurities into the cationic sites of the Ti^4+^ sublattice of the TiO_2_ matrix, leading to the appearance of M^2+^ centres, corresponds with the formation of a pair of substitutional (M_Ti_) and interstitial (M_i_) impurities as follows:

1 $$2\text{Ti}\text{O}_{2} + 2\text{M} \rightarrow \text{M}_\text{Ti}\text{M}_\text{i}\text{O}_{4} + {2\text{Ti}},$$

A relatively large concentration of metal impurities with a low energy cost for incorporation into the interstitial void is essential for this configuration to form. This is not the case for the iron in bulk anatase TiO_2_, where the formation of interstitial impurities is strongly energetically unfavourable^40^. Experimental and theoretical results demonstrate that the most energetically favourable configuration of iron impurities is in the pair of substitutional defects near an oxygen vacancy in bulk TiO_2_. Two equations can describe the required transformation of the local chemical structure when a single oxygen vacancy is introduced:

2 $$2\text{Ti}\text{O}_{2} + 1/2\text{O}_{2} \rightarrow \text{Ti}_\text{2}\text{O}_\text{3},$$

3 $${\text{Ti}}_{2}\text{O}_{3} + 2\text{Fe} \rightarrow \text{Fe}_\text{2}\text{O}_\text{3}+2\text{Ti},$$

The presence of a local Fe_2_O_3_ configuration corresponds with the appearance of Fe^3+^ centres in the TiO_2−x_ matrix. Incorporation of the iron impurities into the interstitial void maximises the possible oxidation states on the metal centre. Our previous work demonstrates experimentally and theoretically the favourability of incorporating cobalt impurities into interstitial voids in anatase TiO_2_ to form Co^3+^centres^42^. The interstitial cobalt impurities form three covalent bonds with the nearest oxygen atoms, causing a reduction of the nearest titanium atoms from Ti^4+^ to Ti^3+^. Thus, the observation of Fe^2+^ and/or Co^2+^ impurities in TiO_2_ nanoparticles with low impurity concentrations can be considered to result specifically from the under-coordination of impurities in the surface layer. On the other hand, relatively high impurity concentrations can lead to the appearance of a detectable number of M^3+^ centres in TiO_2 _nanoparticles^11^.

Previous theoretical calculations have discussed only substitutional impurities in a single selected surface of anatase TiO_2_^20,34,43^or the catalytic properties of nano-clusters on selected surfaces^24,44,45^. As TiO_2_ nanoparticles have different types of facets, the exposure of the preferable surface for the incorporation of impurities is essential to the description of the photocatalytic properties. The effect of local lattice distortions should also be considered, as nanoparticles can have multiple edges and cracks. Thus, a systematic study of the synergetic effects of impurity type, surface, oxygen vacancy, and lattice distortions is essential for understanding the physical and chemical properties of TiO_2_ nanoparticles and their guided design with control over the optical and catalytic properties.

In this work, we report the results of the simulation of incorporating substitutional and interstitial impurities with the pairs of defects in (110), (101), and (100) surfaces of anatase titania with (TiO_1.97_) and without (TiO_2_) oxygen vacancies. We consider doping with both iron and cobalt impurities, as in bulk anatase TiO_2_ they exhibit opposite tendencies towards substitutional location inside the oxide host. To further explore the effect of large defects, such as facets, edges, and surface cracks, we also simulate incorporation of the most energetically favourable defects into strained surfaces. Finally, the simulation of the hydrogen evolution reaction (HER) in acidic media has been carried out for all considered model structures as numerous experimental works have reported a good catalytic performance of TM-doped TiO_2 _nanoparticles^1–3,6,21,24–27,34^. Photo-catalyzed HERs over TiO_2_-based systems have also been reported (see the review by J. Pan et al^46^.). Note that this reaction can also be considered as the step corresponding with the temporal adsorption of hydrogen on the catalytic substrate in other reactions involving doped TiO_2_ as a photo-catalyst.

**Figure 1:**
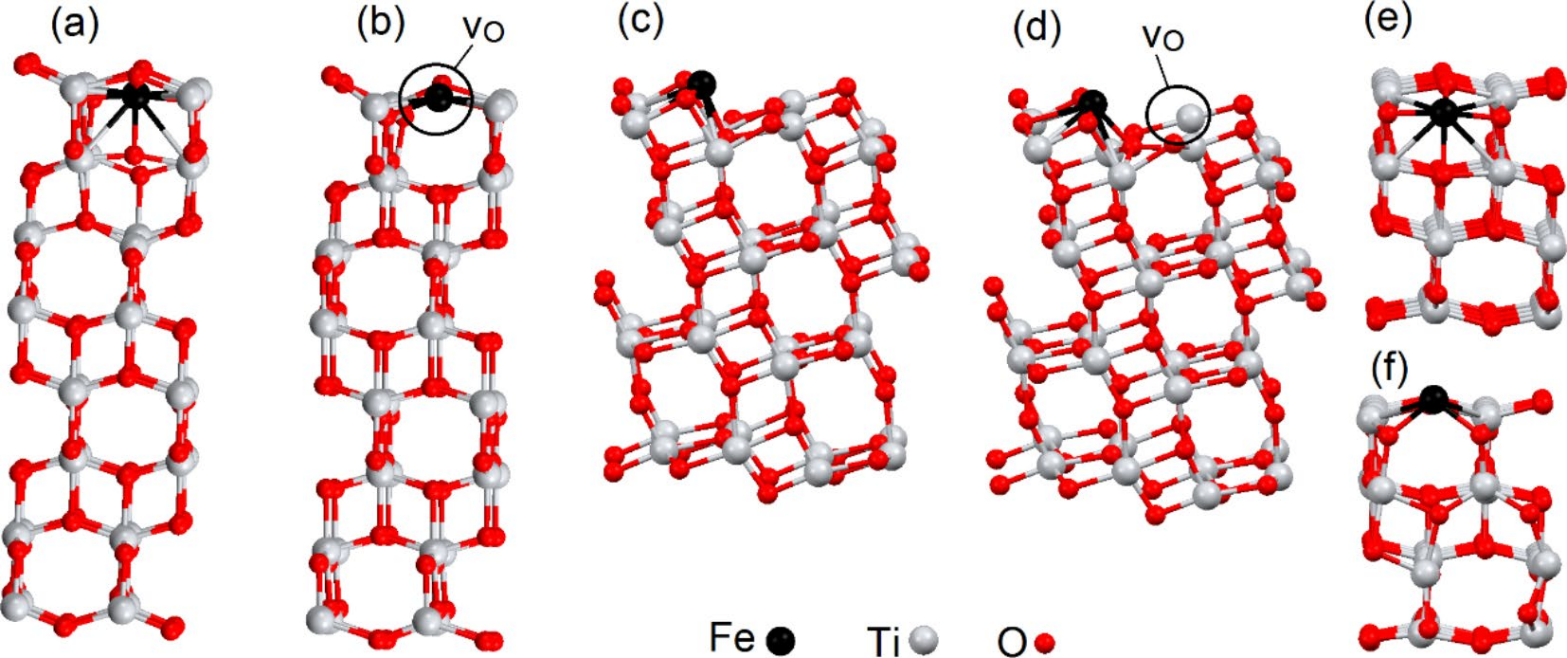
Optimized atomic structure of an interstitial iron impurity (Fe_i_) incorporated into the (110) (a, b), (101) (c, d) and (100) surfaces (e, f) of TiO_2_ (a, c, e) and TiO_1.97_ (b, d, f).

## Computational details

Theoretical modelling was carried out using the SIESTA pseudopotential code^47^ previously used by the authors to simulate doped TiO_2_^41,42^. The generalized gradient approximation with a Perdew–Burke–Ernzerhof functional (GGA-PBE)^48^for the exchange-correlation potential in a spin-polarized mode was employed for the simulations. Using hybrid functionals or DFT + U methods will provide incorrect results as the effects of crystal field, under-coordination, and asymmetry of the local environment lead to a more significant slit of occupied and unoccupied orbitals^49^. A comparison of calculated and experimental spectra for surface impurities in oxides (including TiO_2_) also demonstrates the feasibility of GGA in describing the electronic structure and magnetic properties of the studied systems^41,42,50^. In addition, the values of the bandgap in the systems with surface impurities are determined by defect-related states. The GGA-PBE method is feasible to simulate the discussed systems as the electronic structure of impurities is mainly determined by the local crystal field.

Slabs within periodic boundary conditions constructed from 96 atoms corresponding with different surfaces of TiO_2_ were used in the simulation. These slabs were constructed by multiplication of the unit cell of anatase TiO_2 _obtained via optimization of lattice parameters and atomic positions. The separation between the slabs along the vertical axis was 4 nm. A full optimization of the atomic positions at fixed lattice parameters was carried out in these slabs. The electronic ground state was consistently found using norm-conserving pseudopotentials^51^ for the cores with a double-*ξ*-plus polarization basis for the localized orbitals of non-hydrogen atoms and a double-*ξ* for hydrogen atoms. The energy cut-off was 400 Ry. The forces and total energies were optimized with an accuracy of 0.04 eV Å^−1^ and 1.0 meV/cell (or less than 0.02 meV/atom), respectively. All calculations were conducted with an 8 × 8 × 1 Monkhorst-Pack *k*-point grid for the Brillouin zone sampling^52^. The magnetic moments and, hence, spin-states were explored by subtraction of occupancies of the orbitals with different spins.

Formation energy was calculated by the standard formula:

4 $${E}_{form} = [E(\text{Ti}_\text{x}\text{M}_\text{k}\text{O}_\text{y}) - \{E(\text{Ti}_\text{x-n}\text{O}_\text{z})-{nE}({\text{Ti}})+kE\text({\text{M})}\}]/k,$$

where *E*(Ti_x-n_O_y_) and *E*(Ti_x_M_k_O_y_) are the total energies of the supercell before and after incorporation of *k* impurities, with *n* being the number of substitutional defects. In the case of a single interstitial impurity, *n* equals zero. *E*(Ti) and *E*(M) are the total energies per atom of metallic titanium and incorporated atoms respectively. The enthalpy of the hydrogen atoms chemisorption is defined as:

5 $$\Delta H= E(\text{Ti}_\text{x}\text{M}_\text{k}\text{O}_\text{y}\text{H})-{(E(\text{Ti}_\text{x}\text{M}_\text{k}\text{O}_\text{y})+1/2E({\text{H}_{2}}})],$$

where *E*(Ti_x_M_k_O_y_H) is the total energy of the supercell with a hydrogen atom adsorbed on the metal centre, and *E*(H_2_) is the total energy of a single non-interacting hydrogen molecule. The calculations of the free energy of the HER were performed while taking into account zero-point energy and entropy correction within the standard framework^53^ by the following formula:

6 $$\Delta\text{G}=\Delta{H}+\text{Z}\text{P}\text{E}+\Delta ST,$$

where ZPE is the zero-point energy, and Δ*ST* is the contribution from the entropy change at room temperature. The reference potential is set as zero. We exclude the effect of the solvent from consideration as the objective of our work is to study the influence of different properties of the TiO_2_ substrate on catalytic properties.

## Results and discussion

### Incorporation of metal impurities in unstrained surface

We first calculated the formation energies for various configurations of substitutional (in the Ti^4+^ cation sublattice) and interstitial iron and cobalt impurities in three different surfaces of anatase TiO_2_, with and without oxygen vacancies (see Fig. 1). The thickness of the slabs is about 1.2 ~ 1.6 nm. This thickness is sufficient to correctly estimate the bandgap^54^. Our previous work demonstrated the favourability of the location of Fe and Co defects near oxygen vacancies in TiO_2_^40,41^. Our calculations also demonstrate the favourability of forming oxygen vacancies in surface layers for all studied facets. Thus, we will simulate the location of the atoms near the surface oxygen defects. We simulated oxygen vacancies and defects only in one side of the slabs as the size of TiO_2_ nanoparticles is usually several times larger than the slab thicknesses. Our simulations considered supercells with one or two impurities in surface layers. This amount of impurities corresponds with a concentration of around 2 ~ 4 wt%, which is representative of the wt% reported in the experimental studies discussed in the introductory section.

The formation of single substitutional impurities (in the Ti^4+^ cation sublattice) in the surface and subsurface layers, pairs of substitutional impurities, single interstitial impurities, and paired substitutional and interstitial impurities were considered. The calculated energies are reported in Table 1. The data for E_form_ shown in Table 1 demonstrates that the formation of substitutional defects is energetically favourable for iron impurities and unfavourable for cobalt impurities. Note that only for the case of substitutional iron impurities in the (110) surface, the formation energy for localisation within the subsurface layer is about 0.12 eV lower than in the surface layer. The location in the surface layer is more energetically favourable for all other impurities. The calculated energies for forming pairs of substitutional impurities demonstrate the same pattern. The most energetically favourable configuration for both iron and cobalt incorporation is a single interstitial impurity embedded in the surface. The presence of oxygen vacancies does not change this pattern. Note that formation energies were calculated using the total energies of the metals in the bulk phase. Thus, we can propose that incorporating a visible amount of metal atoms from the nearest metallic surfaces (other nanoparticles, contacts) into the surfaces of TiO_2_ is very probable.

**Table 1:** The calculated formation energies (E_form_), magnetic moments on atoms (M), bandgaps, and differential free energies of hydrogen adsorption (ΔG) for different combinations of substitutional (M_Ti_) and interstitial (M_i_) iron and cobalt impurities incorporated in different surfaces of TiO_2_ and TiO_1.97_ (number in parenthesis). The numbers corresponding with the most prominent configurations for HER are shown in bold.

Surface	Defect	Metal	E_form_, eV/atom	M, *µ*_B_	Bandgap, eV	ΔG, eV/H^+^
110	M_Ti_	Fe Co	−0.07 (− 1.26) + 5.89 (+ 4.72)	4.12 (4.30) 3.30 (3.23)	1.76 (1.87) 0.43 (1.93)	--- + 2.83 (+ 3.15)
	2M_Ti_	Fe Co	−1.09 (− 0.99) + 5.95 (+ 4.66)	4.20, 3.62 (4.30, 4.27) 3.32, 3.31 (3.33, 3.26)	1.57 (2.12) 0.74 (1.95)	--- −1.78 (+ 1.48)
	M_i_	Fe Co	−5.73 (− 2.98) −1.41 (− 1.66)	4.14 (0.04) 2.88 (0.35)	0.92 (1.17) 1.04 (1.16)	+ 0.35 (− 2.66) + 2.18 (− 1.86)
	M_Ti_+M_i_	Fe Co	−**3.83** (− 3.14) + 1.27 (+ 1.84)	4.20, 1.12 (3.63, 4.27) 2.90, 0.08 (2.77, 2.91)	1.13 (0.98) 1.29 (0.79)	**−0.06** (− 0.80) + 0.54 (− 0.89)
101	M_Ti_	Fe Co	−0.31 (− 1.61) + 6.05 (+ 3.63)	3.64 (4.21) 3.30 (2.87)	1.30 (2.10) 0.45 (1.23)	+ 1.51 (− 0.36) + 1.62 (+ 0.99)
	2M_Ti_	Fe Co	−0.15 (− 1.38) + 6.13 (+ 4.34)	3.62, 3.77 (4.21, 4.24) 3.26, 3.37 (3.22, 3.27)	1.87 (1.78) 0.16 (0.17)	+ 1.56 (− 0.55) + 1.49 (− 1.56)
	M_i_	Fe Co	−4.53 (− **5.18**) −0.24 (− 0.83)	3.82 (3.83) 2.90 (2.89)	0.67 (0.85) 1.12 (1.26)	+ 0.46 (− **0.05**) + 0.59 (+ 0.91)
	M_Ti_+M_i_	Fe Co	−**3.14** (− **3.43**) + 1.81 (+ 1.36)	3.92, 4.24 (3.82, 4.19) 2.88, 2.90 (2.91, 2.86)	0.62 (0.16) 0.34 (1.23)	**+ 0.12** (− **0.02**) + 0.77 (+ 0.91)
100	M_Ti_	Fe Co	−0.38 (− 1.67) + 5.82 (+ 4.04)	3.66 (4.22) 2.78 (3.22)	1.91 (1.52) 0.35 (0.78)	+ 1.41 (+ 0.71) + 1.14 (+ 1.20)
	2M_Ti_	Fe Co	−0.19 (− 1.16) + 5.93 (+ 4.52)	3.65, 3.82 (4.17, 4.27) 2.73, 3.32 (3.18, 3.29)	1.45 (2.12) 0.33 (2.16)	+ 1.26 (+ 0.38) + 1.37 (+ 1.14)
	M_i_	Fe Co	−**5.05** (− 5.74) −0.78 (− 1.35)	3.90 (3.91) 2.90 (2.92)	0.62 (0.54) 0.84 (1.49)	**+ 0.15** (+ 0.41) + 0.85 (+ 1.02)
	M_Ti_+M_i_	Fe Co	−**3.27** (− 3.82) + 1.71 (+ 1.05)	3.86, 4.26 (4.17) 2.89, 2.91 (3.83)	0.43 (1.35) 1.23 (1.10)	**+ 0.18** (+ 0.60) + 0.86 (+ 1.20)

This situation is completely different to the incorporation of iron and cobalt into bulk TiO_2_, discussed in the introduction, where iron impurities prefer to occupy substitutional positions in the vicinity of oxygen vacancies^41^. The presence of a substitutional impurity near an interstitial one significantly decreases the energetic favourability for the incorporation of iron impurities. For cobalt, the formation of this type of defect is energetically unfavourable. The presence of oxygen vacancies does not change this pattern. Therefore, at low concentrations of TM impurities in TiO_2_ nanoparticles, the isolation of paired substitutional and interstitial impurities on the surface is highly improbable, such that single interstitial impurities are the predominant defect type. Note that the coordination of interstitial impurities incorporated in the surface layer of titania is lower than for those incorporated in the bulk. This result agrees with previous conclusions regarding the under-coordination of TM impurities in TiO_2 _nanoparticles^20–22^.

**Figure 2:**
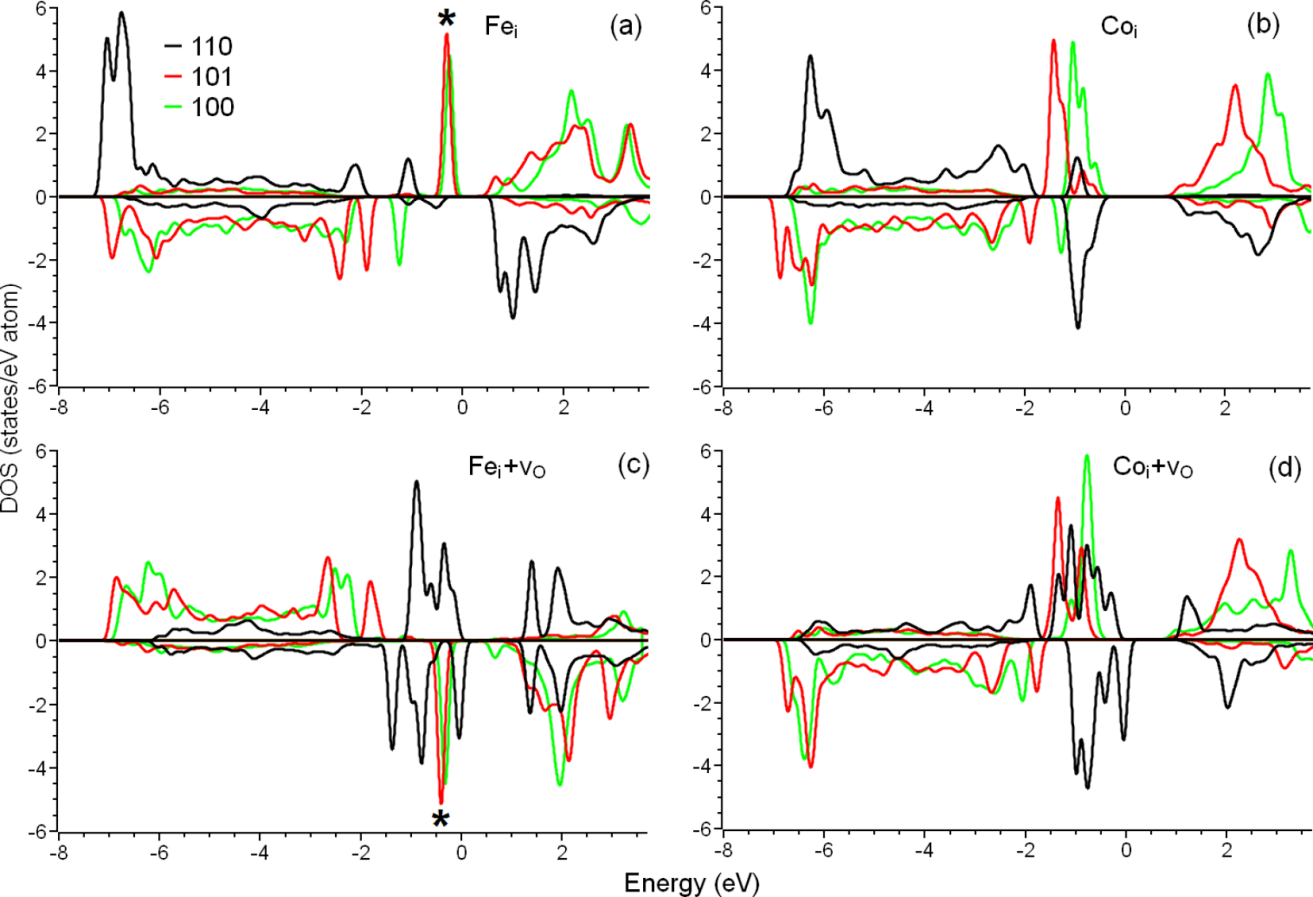
The densities of states of interstitial iron (a, c) and cobalt (b, d) impurities incorporated in different surfaces of TiO_2_ (a, b) and TiO_1.97_ (c, d). The atomic structures of the systems corresponding with (a) and (c) are shown in Fig.  1 . The Fermi energy is set as zero. The distinct peaks that can be associated with high catalytic performance for the hydrogen evolution reaction are marked by asterisks.

The presence of oxygen vacancies in bulk TiO_2_ changes the oxidation state of metal impurities and also, therefore, their corresponding magnetic moments. However, for iron and cobalt defects on the surface, the presence of an oxygen vacancy in the vicinity does not significantly change the magnetic moment of the impurity. The sole exception to this observation is for an interstitial impurity in (110) surface. The magnetic moments on iron and cobalt impurities are 4.14 *µ*_B_ and 2.88 *µ*_B_, respectively, with no oxygen vacancy, whereas with an oxygen vacancy, the local atomic structure changes drastically (see Fig. 1a, b). If the metal impurity is located in the centre of a square planar configuration. This coordination usually corresponds with a low-spin magnetic configuration^49^, and this case does not prove an exception. The magnetic moments of iron and cobalt atoms in these cases decrease drastically (to 0.04 *µ*_B_ and 0.35 *µ*_B_ for iron and cobalt, respectively). This transition from a high to low spin configuration can also be seen clearly in the changes in the spin-resolved density of states (see the difference in the black lines between Fig. 2a and c or Fig. 2b and d). Thus, the main effect of the oxygen vacancies in bulk is to change the oxidation state of impurities with a resulting change to the magnetic properties of impurities, whereas the main effect of oxygen vacancies on the surface is to change the crystal field by introducing additional distortions of the lattice.

**Figure 3:**
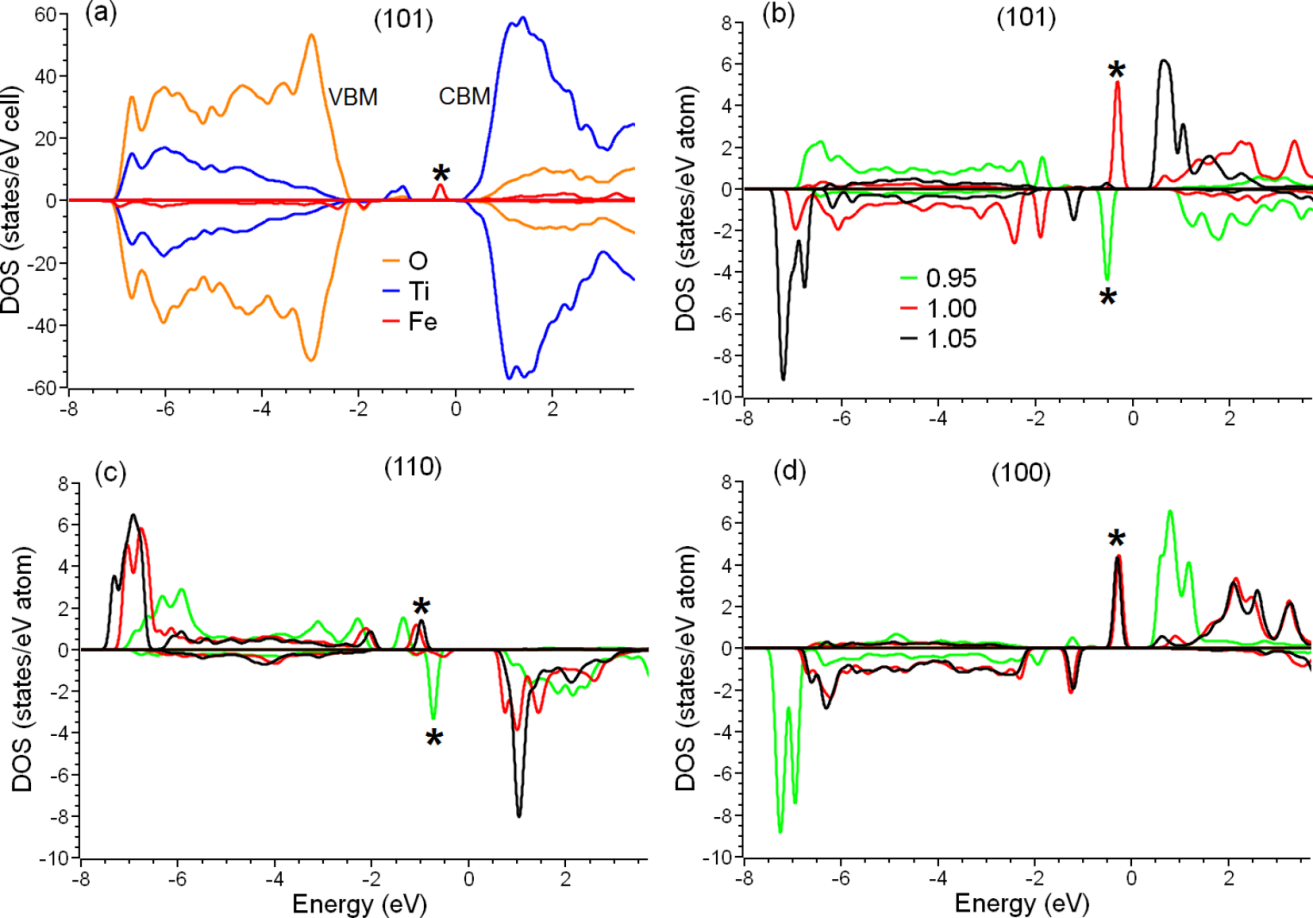
The total densities of states of interstitial iron, titanium, and oxygen atoms for (101) surface of TiO_2_ (a). The densities of states for interstitial iron impurities incorporated into compressed (a/a_0_  = 0.95), unstrained (a/a_0_  = 1.00), and expanded (a/a_0_  = 1.05) surfaces of TiO_2_ for the (101) surface (b), the (110) surface (c) and the (100) surface (d). The Fermi energy is set as zero. Asterisks mark distinct peaks associated with high catalytic performance for HER.

The incorporation of the impurities into the TiO_2_ matrix leads to a shrinking of the calculated bandgap due to the existence of defect states between the valence and conductive bands (see, for example, Fig. 3a). This result is in qualitative agreement with experimental observations of a red shift in TM-doped TiO_2 _nanoparticles. Note that standard DFT-GGA calculations generally underestimate the value of the bandgap^55–57^. In the case of TiO_2 _surfaces, the measured value of the bandgap is 3.4 eV^58^, and the calculated value is 2.5 eV. The calculated values can be easily transformed to their ‘real’ counterparts by the semi-empirical formula built based on the data reported in^57^:

7 $${E}_{gap}=1.1\cdot{E}_{form\,DFT}+0.8\,\text{e}\text{V}.$$

From Table 1, we can see that the formation of defect states inside the bandgap of pure TiO_2_ (see Fig. 3a) leads to a decrease in the bandgap to the range of 1.1 eV to 2.1 eV, which by applying Eq. (7) gives corresponding estimated values from 2.0 eV to 3.1 eV. Experimentally measured decreases in the bandgap of Fe-doped TiO_2 _nanoparticles have also been reported, from 3.4 eV in undoped nanoparticles^58^to 2.4 eV^16^or ~ 3.0 eV^10^. However, the exact value of the bandgap varies significantly for different combinations of the type of surface, specific impurity, and local distortions caused by vacancies and strain (see next section). In other words, the same substitutional iron or cobalt impurities in different facets of the same TiO_2_ nanoparticles result in different changes in the bandgap. This finding explains the diversity of optical properties exhibited by previously reported Fe- and Co-doped TiO_2 _nanoparticles^8,10,11,13,15,17^. Note that the presence of oxygen vacancies provides only tiny changes in electronic structure except for in the case (described above) of interstitial impurities in the (110) surface.

Next, we evaluated the catalytic efficiency of iron and cobalt-doped surfaces of TiO_2_. The highest catalytic performance is associated with the values of the free energy of adsorption (Δ*G*) close to zero. To simulate the intermediate step of this reaction, we placed hydrogen atoms over metal centers and then optimized the atomic positions. The calculated values of the free energies for hydrogen adsorption on the metal centres are reported in Table 1. In most cases, the magnitude of the adsorption energies for cobalt is above 1 eV. This result agrees with previous DFT calculations for hydrogen adsorption to substitutional cobalt in TiO_2 _surfaces^20^. The smallest magnitude value is 0.54 eV, which corresponds with hydrogen adsorption on an interstitial cobalt atom belonging to a Co_Ti_ + Co_i_ pair in the (110) surface. However, the formation energy for this pair is + 1.27 eV per cobalt atom, making this configuration energetically unfavourable. In contrast to cobalt impurities, several configurations of iron impurities in the surface demonstrate the same magnitude of hydrogen adsorption energy as reported for platinum (about 0.1 eV^53^). All these configurations (Fe_Ti_+Fe_i_ in all surfaces of TiO_2_ and in the (101) surface of TiO_1.97_, and Fe_i_ in the (100) surface of TiO_2_ and the (101) surface of TiO_1.97_) have corresponding negative formation energies. Oxygen vacancies near impurity sites lead to clear changes in hydrogen adsorption energies caused by the changes in the electronic structure (a more detailed discussion is included in Sect. 3.3) and changes in the energy costs of substrate distortion caused by the formation of metal-hydrogen bonds. These results explain the lower catalytic activity of Co-doped TiO_2_ nanoparticles and the catalytic efficiency for the HER of Fe-doped TiO_2 _nanoparticles reported in publications^20,21,34,38^. Thus, we can conclude that the combination of the dopant specie and the facet of the nanoparticles are defining factors for the catalytic activity of TM-doped TiO_2_.

### Effect of surface strain on the properties of metal-doped titania surfaces

In the previous section, we discussed how structural distortions caused by oxygen deficiency can significantly affect the photonic, magnetic, and catalytic properties of doped TiO_2_. However, oxygen deficiencies are not the only cause of structural distortions in TiO_2_ nanoparticles. Straining of the crystal lattice provides another route to decreasing the surface energy. The boundaries between facets are a source of surface tension. Thus, the effect of strain on the physico-chemical properties of doped TiO_2_ should also be considered. To do so, we uniaxially expand and compress the three slabs used to model each of the different surfaces. We consider the simulation of interstitial impurities to be the most energetically favourable. We consider rather large (5%) increases or decreases of the lattice parameter in the ground state (a_0_) to clearly demonstrate the effects produced. Such distortions can be experimentally obtained in TiO_2 _nanoparticles under a pressure of 30 GPa^59^. This significant strain could also be a useful model for the local lattice distortions in the vicinity of the facet’s edges or larger structural defects such as cracks. The absence of any observed effects on the physical properties from the lattice strain, even at the large deviations of lattice parameters used, is also evidence for the stability of these properties at smaller lattice strains.

**Table 2:** The calculated formation energies (E_form_), magnetic moments on atoms (M), bandgaps, and differential free energies of hydrogen adsorption (ΔG) for interstitial (M_i_) iron and cobalt impurities incorporated in different surfaces of TiO_2_ and TiO_1.97_ (numbers in parentheses) with different ratios of in-plane lattice constants (a) to the value of the lattice constant in ground state (a_0_).

Impurity	Surface	a/a_0_	E_form_, eV	M, *µ*_B_	Bandgap, eV	ΔG, eV/H^+^
Fe	110	1.05 1.00 0.95	−6.90 (− 6.19) −5.73 (− 2.89) −4.30 (− 5.50)	4.26 (4.08) 4.14 (0.04) 3.88 (4.01)	1.16 (1.61) 0.92 (1.17) 1.34 (1.09)	+ 1.50 (− 0.77) + 0.35 (− 2.66) + 3.33 (+ 2.78)
	101	1.05 1.00 0.95	−5.97 (− 5.49) −4.35 (− **5.18**) −**4.70** (− 4.42)	4.21 (3.84) 3.82 (3.83) 3.88 (3.84)	0.88 (1.06) 0.67 (0.85) 0.98 (1.02)	+ 0.51 (+ 0.15) + 0.46 (− **0.05**) **+ 0.01** (− 0.53)
	100	1.05 1.00 0.95	−5.90 (− 5.78) −**5.05** (− 5.74) −6.53 (− 2.87)	3.91 (4.15) 3.90 (3.91) 4.20 (0.94)	0.66 (1.08) 0.62 (0.54) 0.95 (1.32)	+ 0.25 (+ 1.02) **+ 0.10** (+ 0.41) + 1.08 (+ 1.76)
Co	110	1.05 1.00 0.95	−1.99 (− 1.32) −1.41 (− 1.61) −1.21 (− 2.21)	2.91 (2.89) 2.88 (0.35) 2.86 (2.79)	0.90 (1.34) 1.04 (1.16) 1.33 (1.18)	+ 0.52 (− 0.79) + 2.18 (− 1.86) + 1.02 (− 0.71)
	101	1.05 1.00 0.95	−1.37 (− 1.08) −0.24 (− 0.83) −**0.26** (− 0.99)	2.90 (2.91) 2.90 (2.89) 2.92 (1.32)	1.20 (1.30) 1.12 (1.26) 1.50 (0.92)	+ 0.73 (+ 0.69) + 0.59 (+ 0.91) **+ 0.10** (− 1.79)
	100	1.05 1.00 0.95	−1.61 (− 1.20) −0.78 (− 1.35) −1.46 (− 0.57)	2.89 (2.93) 2.90 (2.92) 2.93 (2.91)	1.01 (1.24) 0.84 (1.49) 0.91 (1.05)	+ 1.03 (+ 0.99) + 0.85 (+ 1.02) + 0.99 (+ 1.25)

The formation energies for the interstitial defects have been calculated (Table 2), demonstrating observable changes to the formation energies without changing the overall patterns between values for different combinations of impurities and surfaces. Since the magnitudes of the formation energies are relatively high (especially for iron), some decreases in the values do not significantly affect the stability of the discussed defects. Favourability of the incorporation of TM impurities in strained TiO_2_ should reduce favourability for the segregation of TM impurities outside of the surface on the edges of facets and cracks in the nanoparticles or grain boundaries in the films. Formation of an interface between metallic and TiO_2_ nanoparticles can also induce strain in the surface area of titania. Thus, the migration of some atoms from metallic nanoparticles into the surface of TiO_2_ could be proposed.

The results of the calculations demonstrate the significant influence of the surface strain on electronic structure for several M_i_:TiO_2_ cases. For example, expanding the slab corresponding to the (101) surface leads to the transformation of the metal centre from Fe^2+^ to Fe^3+^, due to the formation of the additional Fe-O bond. This change is clearly seen in the elimination of the distinct peak below the Fermi level (see Fig. 3b) and the increase of the magnetic moment from 3.82 *µ*_B_ to 4.21 *µ*_B_. In the case of iron and cobalt impurities in the (110) surface of TiO_1.97_, in-plane distortions of the lattice lead to the transition from the low-spin configurations shown in Fig. 2c, d to high-spin configurations with a corresponding increase in the magnetic moment (see Table 2). Thus, one can say that lattice strain can counteract the effect of the local distortions caused by oxygen vacancies. Contrary to the cases described above, compression of the (100) surface of Fe_i_:TiO_1.97_ and the (101) surface of Co_i_:TiO_1.97_ switches the magnetic configuration of the impurities from high-spin to low-spin (see Table 2). The influence on the magnetic properties of the dopants is insignificant (about 0.2 *µ*_B_) for other combinations of defects, vacancies, surfaces, and strains.

**Figure 4:**
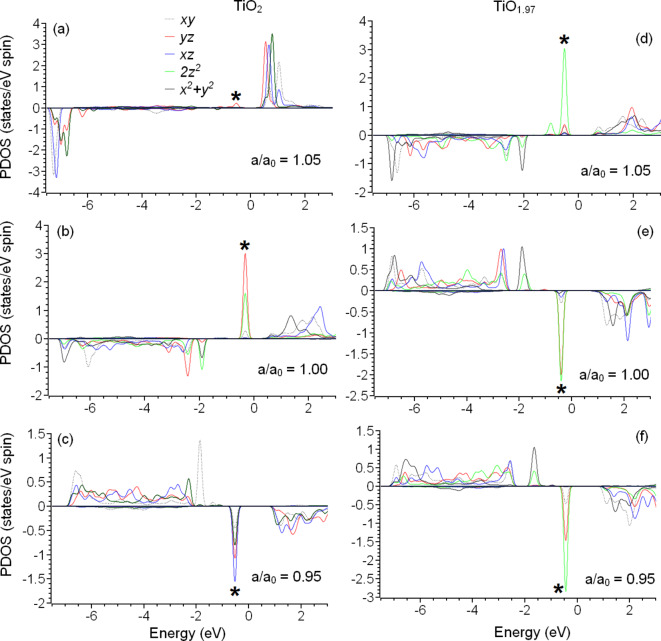
The partial spin-polarized densities of states for 3*d* orbitals of interstitial iron impurities in strained and unstrained surfaces of TiO_2_ (a-c) and TiO_1.97_ (d-f). The Fermi energy is set as zero. Asterisks mark distinct peaks associated with high catalytic performance for the hydrogen evolution reaction.

The effect of the surface strain on the calculated bandgaps is also visible. Compression or expansion of the surface layers leads to changes of the calculated values of the energy gap by 0.3 ~ 0.5 eV. Note that—with a few exceptions—lattice strain leads to an increase of the bandgap. To understand the nature of this effect, the electronic structure of the TM impurities should be analysed. The typical electronic structure of the valence bands of TM oxides consists of 3*d* bands and oxygen 2*p *bands below the Fermi level^60,61^. This rule also holds for cluster structures based on metal oxides^62^. Under-coordination of the surface impurities makes the electronic structure of the 3*d* orbitals of metals more atom-like, with a larger part of the 3*d* bands lying between − 3 eV and − 8 eV, often with a distinct density-of-state peak at − 7 eV on the lower edge of the O 2*p* band (see Fig. 2). This is the reason why DFT + U methods are excessive for the considered systems. Only single, narrow, distinct peaks located between O 2*p* bands and Fermi level, as is typical for 3*d* bands, is observed (peaks are marked by asterisks in Figs. 3 and 4). Expanding the surface makes the electronic structure of the metal impurities even more atom-like due to the increased length of the metal-oxygen bonds, which leads to the bandgap increasing. On the other hand, compression of the surface increases local distortions and, according to ligand field theory, decreases the energy levels of the occupied orbitals^62^. Since nanoparticles of different sizes have different magnitudes of surface tension, the diversity of the optical absorption and fluorescent properties of nanoparticles with different sizes can be suitably explained as an effect of surface mechanical distortions on the electronic structure of metal centres.

The most significant effect of substrate strain is on the catalytic properties of metal centres. The magnitudes of the free energies for the free energy of hydrogen adsorption changed in all considered configurations. In several cases, even a change of the sign of the free energies was observed (for example, compression of (110) surface of Fe_i_:TiO_1.97_ leads to a change of ΔG from − 2.66 eV/H^+^ to + 2.78 eV/H^+^). For the compressed (101) surface of Co_i_:TiO_2_, the magnitude of the free energy approaches very close to that of platinum (about 0.1 eV^53^). However, the corresponding formation energy of this defect is rather high (− 0.26 eV), and the compression of 5% is substantial; thus, this case can be described as hypothetical. Since the calculated magnitudes of the free energies of hydrogen adsorption on cobalt centres remain quite large, one can conclude that lattice distortions do not improve the catalytic properties of Co-doped TiO_2_.

Strains of the (110) and (100) surfaces of iron-doped TiO_2_ increase the magnitude of the free energies of hydrogen adsorption, which corresponds with worse catalytic properties. On the other hand, the energy costs of the HER over the (101) surfaces of TiO_2_ and TiO_1.97_ are not significantly affected by strains. Even the largest magnitudes of the free energies remain within a reasonable range (about 0.5 eV). Note that the (101) surface is the most frequently observed facet in TiO_2 _nanoparticles^40^. Thus, the numerous reports of good catalytic performance of iron-doped TiO_2_ can be attributed to interstitial iron impurities incorporated in the surface of this facet.

### Relationships between electronic structure and catalytic properties

In the previous section, we demonstrated that the best catalytic performance for the HER corresponds with interstitial iron impurities embedded in the (101) surface of TiO_2_ and TiO_1.97_. The main similarity between all six configurations of the two substrates and three types of lattice distortions is the presence of a distinct density-of-states peak below the Fermi level (marked by an asterisk in Fig. 4); thus, the catalytic activity of the considered structures can be inferred as mainly associated with this peak. Note that the presence of this peak is observed in both the strained and unstrained (101) surfaces with and without oxygen vacancies. Therefore, the stability of this peak to local structural distortions is the crucial factor for the high catalytic activity of iron-doped TiO_2_. However, the magnitudes of the calculated free energies of hydrogen adsorption on compressed Fe_i_:TiO_2_ and unstrained Fe_i_:TiO_1.97_ are lower than those for platinum (0.01 eV and 0.05 eV vs. 0.1 eV) and one order of magnitude smaller than for the other structures considered in this section. Therefore, we can ascertain that some other hidden factors not analysed herein also influence the catalytic performance of the discussed structures.

To understand the intricacies of the relationships between electronic structure and catalytic properties of the (101) surface of Fe_i_:TiO_2_ and Fe_i_:TiO_1.97_, we visualised the partial densities of states (PDOS) with and without strains. In the case of expanded Fe_i_:TiO_2_ with a 3*d*^*5*^ configuration, the minor peak below the Fermi level corresponds to the *yz* orbital (see Fig. 4a). Other orbitals demonstrate a minuscule overlap with the O 2*p* orbitals (see Fig. 3a). Thus, the interstitial iron atom in the (101) surface of TiO_2_ has more similarities with a Fe^3+^ ion in empty space than with an iron impurity embedded in some matrix. In unstrained Fe_i_:TiO_2_ with a 3*d*^*6*^ configuration, the distinct energy peak at − 0.5 eV derives from the contributions of the *yz* and 2*z*^*2*^ orbitals (see Fig. 4b). The compression of the TiO_2_ matrix leads to a significant redistribution of the occupancies (population) of the 3*d* energy levels of the iron impurity, such that the distinct peak at − 0.25 eV then consists of visible contributions from all five 3*d* orbitals. The most significant contribution to this energy peak is from the *x*^*2*^ *+ y*^*2*^ type orbital (see Fig. 4c). Note, that switching from lattice expansion to lattice compression also increases the overlap between the Ti 3*d* and O 2*p* orbitals, which corresponds with an increase in the densities of states in the interval − 2…−6 eV.

The main difference between strain-induced changes in the electronic structures of Fe_i_:TiO_2_ and Fe_i_:TiO_1.97_ is the *d*^*6*^ configuration of 3*d* orbital of iron embedded in the oxygen-deficient expanded TiO_2_ matrix. The local distortions caused by the presence of the oxygen vacancy (see Fig. 1c, d) lead to stronger interactions between the 3*d*-metal impurities and the surrounding oxygen atoms and, therefore, the electronic structure of the 3*d* orbitals is closer to the typical electronic structure of a metal impurity than to a free ion (see Fig. 4d vs. Figure 4a). The distinct peak below the Fermi level is associated with an electron with opposite spin to the overall spin of the atom, shifted from − 0.25 eV to − 0.5 eV, and consists almost entirely of the contribution from the 2*z*^*2*^ orbital. The electronic structures of Fe_i_ in unstrained and compressed Fe_i_:TiO_1.97_ matrices are similar to the electronic structures of Fe_i_:TiO_2_ (see Fig. 4e, f). Note that the distinct density-of-states peak at − 0.25 eV also consists of the contribution from different orbitals (predominantly the *xz* and 2*z*^*2*^, see Fig. 4e). The calculated free energies of the HER summarized in Table 2 suggest that the catalytic performance is best for compressed Fe_i_:TiO_2_ and unstrained Fe_i_:TiO_1.97_. In both these structures, the distinct peak below Fermi level consists of almost equal contributions from different orbitals. This makes the electronic structure of the iron centre in stoichiometric or non-stoichiometric titania similar to the electronic structure of the (111) surface of platinum^52^. This electronic structure similarity explains the magnitudes of the free energies for hydrogen adsorption on platinum and the interstitial iron impurity in the (101) surface of titania.

## Conclusions

Based on the results of the calculations, we can claim that interstitial TM impurities in the surface layer are the most energetically favourable defect configurations for the (110), (101), and (100) surfaces of TiO_2_. Neither oxygen vacancies nearby nor significant (5%) surface strain affects the energetic favourability of this type of defect. Thus, the results demonstrate the unfavourability of the segregation of the impurities in the surface. The calculations further demonstrate that incorporation of the impurities into TiO_2_ surfaces always leads to a “red shift”, with the bandgap decreasing to values in the range between 2.0 and 3.1 eV. The exact value of the bandgap depends on the nature of the incorporated species, configuration of defects, and type of surface. The calculated magnetic moment on TM impurities is almost the same for each different facet and, with a few exceptions, is affected neither by surface strain nor by the presence of an oxygen vacancy nearby.

Simulation of in-plane uniaxial lattice distortions and compressions showed that it does not lead to valuable changes in formation energies. Thus, our simulations predict that impurities will be uniformly distributed on the surfaces of TiO_2_ without segregation on facets’ edges or near other large defects such as cracks. Inducing substrate strain leads to a corresponding blue shift at about 0.2 eV. Evaluation of the catalytic performance for the hydrogen evolution reaction in acidic media demonstrates the unsuitability of cobalt-doped TiO_2_ as a catalyst for this reaction. The results of the modelling also demonstrate that local distortions of the atomic structure caused by oxygen vacancies and surface strain do not improve the catalytic performance of cobalt-doped titania. On the other hand, some combination of local distortions of the atomic structure can lead to a redistribution of electron density between the 3*d* orbitals of interstitial iron impurities in (101) surface. These changes in electronic structure lead to a decrease in the magnitude of the calculated free energy of hydrogen adsorption, giving values similar to those of platinum. Based on our results, we can propose that oxygen-deficient iron-doped TiO_2_ nanoparticles of larger size with prevailing (101) surface facets show the best potential for HER in acidic media and other reactions that require the interaction of hydrogen with the catalytic substrate.

Studies of the electronic structure demonstrate the unambiguous association of catalytic activity with the peak on the edge of valence bands. Our comprehensive framework for evaluation of the influence of type of surface, oxygen vacancies, and surface distortions on the favourability of defect configurations, physical, and chemical properties of doped-TiO_2_ can be used for simplification of further predictions towards finding better combinations of dopants and substrate properties (such as size of nanoparticles, damage induced by radiation, and oxygen deficiency) to generate desirable combinations of the bandgap and catalytic/photocatalytic performance. This simplification can be realized by excluding the simulation of substitutional surface and subsurface defects and checking peculiarities in the electronic structure instead of calculating adsorption energy.

## Data Availability

The data that support the findings of this study are available from the corresponding author, Danil Boukhvalov, upon reasonable request.

## References

[1] Kumaravel, V., Mathew, S., Bartlett, J. & Pillai, S. C. Photocatalytic hydrogen production using metal doped TiO_2_: a review of recent advances. *Appl. Catal. B: Envir*. **244**, 1021–1064. 10.1016/j.apcatb.2018.11.080 (2019).

[2] Bakbolat, B. et al. Recent developments of TiO_2_-Based photocatalysis in the Hydrogen Evolution and Photodegradation. *Rev. Nanomaterials*. **10**, 1790. 10.3390/nano10091790 (2020). 10.3390/nano10091790 PMC7558756 32916899

[3] Do, H. H. et al. Recent progress in TiO_2_-based photocatalysts for hydrogen evolution reaction: a review. *Arab. J. Chem.* **13**, 3653–3671. 10.1016/j.arabjc.2019.12.012 (2020).

[4] Varma, K. S. et al. Photocatalytic degradation of pharmaceutical and pesticide compounds (PPCs) using doped TiO_2_ nanomaterials: a review. *Water-Energy Nexus*. **3**, 46–61. 10.1016/j.wen.2020.03.008 (2020).

[5] Ismael, M. A review and recent advances in solar-to-hydrogen energy conversion based on photocatalytic water splitting over doped-TiO_2_ nanoparticles. *Solar Ener*. **211**, 522–546. 10.1016/j.solener.2020.09.073 (2020).

[6] Xia, C. et al. Emerging cocatalysts in TiO_2_-based photocatalysts for light-driven catalytic hydrogen evolution: progress and perspectives. *Fuel*. **307**, 121745. 10.1016/j.fuel.2021.121745 (2022).

[7] Nur, A. S. M. et al. A review on the development of elemental and codoped TiO_2_ photocatalysts for enhanced dye degradation under UV–vis irradiation. J. Water Proc. Engin. 47, 102728. (2022). 10.1016/j.jwpe.2022.102728

[8] Radhika, V. N., Venkata, S. G., Murukeshan, V. M. & Vijayan, C. A review on optical bandgap engineering in TiO_2_ nanostructures via doping and intrinsic vacancy modulation towards visible light applications. *J. Phys. D: Appl. Phys.* **55**, 313003. 10.1088/1361-6463/ac6135 (2022).

[9] Ge, S. et al. A review on the progress of Optoelectronic devices based on TiO_2_ Thin films and nanomaterials. *Nanomaterials*. **13**, 1141. 10.3390/nano13071141 (2023). 37049236 10.3390/nano13071141 PMC10096923

[10] Tayade, R. J., Kulkarni, R. G. & Jasra, R. V. Photocatalytic degradation of aqueous nitrobenzene by nanocrystalline TiO_2_. *Ind. Eng. Chem. Res.* **45**, 922–927. 10.1021/ie051060m (2006).

[11] Asiltürk, M., Sayılkan, F. & Arpaç, E. Effect of Fe^3+^ ion doping to TiO_2_ on the photocatalytic degradation of Malachite Green dye under UV and vis-irradiation. *J. Photochem. Photobiol A*. **203**, 64–71. 10.1016/j.jphotochem.2008.12.021 (2009).

[12] Dholam, R., Patel, N., Adami, M. & Miotello, A. Hydrogen production by photocatalytic water-splitting using Cr-or Fe-doped TiO_2_ composite thin films photocatalyst. *Int. J. Hydrogen Energy*. **34**, 5337–5346. 10.1016/j.ijhydene.2009.05.011 (2009).

[13] Sun, T. et al. Fe and Ni co-doped TiO_2_ nanoparticles prepared by the alcohol-thermal method: application in hydrogen evolution by water splitting under visible light irradiation. *Powder Technol.* **228**, 210–218. 10.1016/j.powtec.2012.05.018 (2012).

[14] Kernazhitsky, L. et al. A comparative study of optical absorption and photocatalytic properties of nanocrystalline single-phase anatase and rutile TiO_2_ doped with transition metal cations. *J. Solid State Chem.* **198**, 511–519. 10.1016/j.jssc.2012.11.015 (2013).

[15] Bhosale, R. et al. Solar photocatalytic degradation of methylene blue using doped TiO_2_ nanoparticles. *Sol Energy*. **103**, 473–479. 10.1016/j.solener.2014.02.043 (2014).

[16] Kamble, R. J. & Gaikwad, P. V. Peroxy titanium complex derived Fe-doped TiO_2_ nanoparticles: synthesis, properties and antibacterial activity. *Mater. Today Pros*. **45**, 3784–3788. 10.1016/j.matpr.2021.01.282 (2021).

[17] Mancuso, A. et al. Visible light active Fe-Pr co-doped TiO_2_ for water pollutants degradation. *Catal. Today*. **380**, 93–104. 10.1016/j.cattod.2021.04.018 (2021).

[18] Bae, J. et al. Efficiency improvement of dye-sensitized solar cells using Cu,Co/TiO_2_ photoelectrodes doped by applying ultrasonic treatment. *Appl. Surf. Sci.* **621**, 156823. 10.1016/j.apsusc.2023.156823 (2023).

[19] Sinhmar, A., Setia, H., Kumar, V., Sobti, A. & Pal Toor, A. Enhanced photocatalytic activity of nickel and nitrogen codoped TiO_2_ under sunlight. *Envir Tech. Innov.* **18**, 100658. 10.1016/j.eti.2020.100658 (2020).

[20] Wu, X. et al. Atomically defined Co on two-dimensional TiO_2_ nanosheet for photocatalytic hydrogen evolution. *Chem. Eng. J.* **420**, 127681. 10.1016/j.cej.2020.127681 (2021).

[21] Xiao, M. et al. Molten-salt-mediated synthesis of an atomic nickel co-catalyst on TiO_2_ for Improved Photocatalytic H_2_ Evolution. *Angew Chem.* **132**, 7297–7301. 10.1002/ange.202001148 (2020). 10.1002/anie.202001148 32067299

[22] Lee, B. H. et al. Reversible and cooperative photoactivation of single-atom Cu/TiO_2_ photocatalysts. *Nat. Mater.* **18**, 620–626. 10.1038/s41563-019-0344-1 (2019). 31011217 10.1038/s41563-019-0344-1

[23] Chang, S. & Liu, W. Surface doping is more beneficial than bulk doping to the photocatalytic activity of vanadium-doped TiO_2_. *Appl. Catal. B: Environ.* **101**, 333–342. 10.1016/j.apcatb.2010.09.035 (2011).

[24] Wang, D. & Gong, X. Q. Function-oriented design of robust metal cocatalyst for photocatalytic hydrogen evolution on metal/titania composites. *Nat. Commun.* **12**, 158. 10.1038/s41467-020-20464-x (2021). 33420037 10.1038/s41467-020-20464-x PMC7794313

[25] Pulido Melián, E. et al. González Díaz, highly photoactive TiO_2_ microspheres for photocatalytic production of hydrogen. *Int. J. Hydrogen Ener*. **44**, 24653–24666. 10.1016/j.ijhydene.2019.07.230 (2019).

[26] Chen, Y. Engineering the Atomic interface with single platinum atoms for enhanced photocatalytic hydrogen production. *Angew Chem. Int. Ed.* **59**, 1295–1301. 10.1002/anie.201912439 (2019). 10.1002/anie.201912439 31654544

[27] Fan, L. et al. Single-site nickel-grafted anatase TiO_2_ for hydrogen production: toward understanding the nature of visible-light photocatalysis. *J. Catal.* **320**, 147–159. 10.1016/j.jcat.2014.09.020 (2014).

[28] Osipov, V. Y. et al. Titanium dioxide nanoparticles heavily doped with niobium: a light-induced electron paramagnetic resonance study. *Mend Commun.* **33**, 349–352. 10.1016/j.mencom.2023.04.017 (2023).

[29] Yamashita, H. et al. Photocatalytic degradation of organic compounds diluted in water using visible light-responsive metal ion-implanted TiO_2_ catalysts: fe ion-implanted TiO_2_. *Catal. Today*. **84**, 191–196. 10.1016/S0920-5861(03)00273-6 (2003).

[30] Nagaveni, K., Hegde, M. S. & Madras, G. Structure and Photocatalytic Activity of Ti_1-x_M_x_O_2±δ_ (M = W, V, Ce, Zr, Fe, and Cu) Synthesized by Solution Combustion Method. J. Phys. Chem. B 108, 20204–20212. (2004). 10.1021/jp047917v

[31] Luttrell, T., Halpegamage, S., Tao, J., Kramer, A. & Sutter, E. Why is anatase a better photocatalyst than rutile? - model studies on epitaxial TiO_2_ films. *Sci. Rep.* **4**, 4043. 10.1038/srep04043 (2014). 24509651 10.1038/srep04043 PMC3918909

[32] Wang, H. X., Li, G. J., Kamiyama, H., Moriyoshi, Y. & Ishigaki, T. Wavelength-sensitive photocatalytic degradation of methyl orange in aqueous suspension over Iron(III)-doped TiO_2_ nanopowders under UV and visible light irradiation. *J. Phys. Chem. B*. **110**, 6804–6809. 10.1021/jp060082z (2006). 16570988 10.1021/jp060082z

[33] Jaimy, K. B., Safeena, V., Ghosh, S., Hebalkar, N. Y. & Warrier, K. Photocatalytic activity enhancement in doped titanium dioxide by crystal defects. *Dalton Trans.* **41**, 4824–4832. 10.1039/C2DT12018F (2012). 22392625 10.1039/c2dt12018f

[34] Zhao, W. et al. Direct microwave–hydrothermal synthesis of Fe-doped titania with extended visible-light response and enhanced H_2_-production performance. *Chem. Eng. J.* **283**, 105–113. 10.1016/j.cej.2015.07.064 (2016).

[35] Garza-Arévalo, J. I., García-Montes, I., Reyes, M. H., Guzmán-Mar, J. L. & Rodríguez-González, V. Hinojosa Reyes, Fe doped TiO_2_ photocatalyst for the removal of as(III) under visible radiation and its potential application on the treatment of As-contaminated groundwater. *Mater. Res. Bull.* **73**, 145–152. 10.1016/j.materresbull.2015.08.034 (2016).

[36] Ambati, R. & Gogate, P. R. Ultrasound assisted synthesis of iron doped TiO_2_ catalyst. *Ultrason. Sonochem*. **40A**, 91–100. 10.1016/j.ultsonch.2017.07.002 (2018). 10.1016/j.ultsonch.2017.07.002 28946502

[37] Valero-Romero, M. J. et al. Photocatalytic properties of TiO_2_ and Fe-doped TiO_2_ prepared by metal organic framework-mediated synthesis. *Chem. Eng. J.* **360**, 75–88. 10.1016/j.cej.2018.11.132 (2019).

[38] Ismael, M. Enhanced photocatalytic hydrogen production and degradation of organic pollutants from Fe (III) doped TiO_2_ nanoparticles. *J. Envir Chem. Eng.* **8**, 103676. 10.1016/j.jece.2020.103676 (2020).

[39] Li, D. et al. Effects of Particle Size on the Structure and Photocatalytic Performance by Alkali-Treated TiO_2_. Nanomaterials 10, 546. (2020). 10.3390/nano10030546 10.3390/nano10030546 PMC7153365 32197421

[40] Rostami, M. et al. Nano-architectural design of TiO_2_ for high performance photocatalytic degradation of organic pollutant: a review. *Envir Res.* **212D**, 113347. 10.1016/j.envres.2022.113347 (2022). 10.1016/j.envres.2022.113347 35513059

[41] Yermakov, A. Y. Formation of stable Fe-Fe antiferromagnetic dimers in doped TiO_2_:Fe nanoparticles. *J. Phys. Chem. C*. **123**, 1494–1505. 10.1021/acs.jpcc.8b10553 (2019).

[42] Yermakov, A. Y. Unconventional magnetism of non-uniform distribution of Co in TiO_2_ nanoparticles. *J. All Comp.* **826**, 154194. 10.1016/j.jallcom.2020.154194 (2020).

[43] Liu, X. et al. A fundamental DFT study of Anatase (TiO_2_) doped with 3d transition metals for high photocatalytic activities. *J. Wuhan Univ. Technol. -Mat Sci. Edit*. **33**, 403–408. 10.1007/s11595-018-1836-5 (2018).

[44] Jung, M. et al. Exploring cu oxidation state on TiO_2_ and its transformation during photocatalytic hydrogen evolution. *Appl. Catal. Gen.* **521**, 190–201. 10.1016/j.apcata.2015.11.013 (2016).

[45] Zhuang, P., Yue, H., Dong, H. & Zhou, X. Effects of a Ni cocatalyst on the photocatalytic hydrogen evolution reaction of anatase TiO_2_ by first-principles calculations. *New. J. Chem.* **44**, 5428–5437. 10.1039/C9NJ06398F (2020).

[46] Pan, J. et al. Recent progress in Photocatalytic Hydrogen Evolution. *Acta Phys. -Chim Sin*. **36** (X), 0001–0009. 10.3866/PKU.WHXB201805068 (2020).

[47] Soler, J. M. et al. The SIESTA Method for Ab-Initio Order-N materials Simulation. *J. Phys. : Condens. Matter*. **14**, 2745. 10.1088/0953-8984/14/11/302 (2002).

[48] Perdew, J. P., Burke, K. & Ernzerhof, M. Generalized gradient approximation made simple. *Phys. Rev. Lett.* **77**, 3865. 10.1103/PhysRevLett.77.3865 (1996). 10062328 10.1103/PhysRevLett.77.3865

[49] Boukhvalov, D. W., Osipov, V. Y., Serikkanov, A. & Takai, K. Unveiling the structure of metal–nanodiamonds bonds: experiment and theory. *C*. **10**, 63. 10.3390/c10030063 (2024).

[50] Leedahl, B. et al. Study of the structural characteristics of 3*d* metals Cr, Mn, Fe, Co, Ni and Cu implanted in ZnO and TiO_2_ – experiment and theory. *J. Phys. Chem. C*. **118**, 28143–28151. 10.1021/jp509761c (2014).

[51] TroullierO.N. & MartinsJ.L. Efficient pseudopotentials for Plane-Wave calculations. *Phys. Rev. B*. **43** (1993). 10.1103/PhysRevB.43.1993 (1991). 10.1103/physrevb.43.1993 9997467

[52] Monkhorst, H. J. & Pack, J. D. Special points for Brillouin-Zone integrations. *Phys. Rev. B*. **13**, 5188. 10.1103/PhysRevB.13.5188 (1976).

[53] Nørskov, J. K. et al. U. Stimming trends in the exchange current for hydrogen evolution. *J. Electrochem. Soc.* **152**, J23. 10.1149/1.1856988 (2005).

[54] Di Liberto, G., Tosoni1, S. & Pacchioni, G. Role of surface termination in forming type-II photocatalyst heterojunctions: the case of TiO_2_/BiVO_4_. *J. Phys. : Condens. Matter 2021*, **33**, 075001. 10.1088/1361-648X/abc357 10.1088/1361-648X/abc357 33086209

[55] Figgis, B. N. & Hitchman, M. A. Ligand Field Theory and Its Applications. Wiley ISBN: 978-0-471-31776-0 (1999).

[56] Cramer, C. J. Essentials of computational chemistry. Theory and Models. Wiley ISBN 0-0470-09181-9 (Chap. 8). (2004).

[57] van Schilfgaarde, M., Kotani, T., Faleev, S., Quasiparticle Self-Consistent, G. W. & Theory *Phys. Rev. Lett.* **96**, 226402. 10.1103/PhysRevLett.96.226402 (2006). 16803332 10.1103/PhysRevLett.96.226402

[58] Reddy, K. M., Manorama, S. V. & Reddy, A. R. Bandgap studies on anatase titanium dioxide nanoparticles. *Mater. Chem. Phys.* **78**, 239–245. 10.1016/S0254-0584(02)00343-7 (2003).

[59] Zhang, X. et al. High-pressure phase transitions in densely packed nanocrystallites of TiO_2_-II. *J. Phys. Chem. C*. **124**, 1197–1206. 10.1021/acs.jpcc.9b09932 (2020).

[60] Brocŀawik, E. Density functional theory and transition metal oxides. *Theor. Comp. Chem.* **2**, 349–370. 10.1016/S1380-7323(05)80040-8 (1995).

[61] Wang, H. et al. The electronic structure of transition metal oxides for oxygen evolution reaction. *J. Mater. Chem. A*. **9**, 19465–19488. 10.1039/D1TA03732C (2021).

[62] Boukhvalov, D. W. et al. Electron correlation effects in band structure of magnetic clusters Mn_12_ and Fe_8_. *J. El Spec. Rel Phen*. (SI), 137–140. 10.1016/j.elspec.2004.02.015 (2004).

